# Geographical variation and area-level socioeconomic disadvantage in recognized depression: A nationwide register-based Danish cohort study

**DOI:** 10.1007/s00127-026-03055-x

**Published:** 2026-04-29

**Authors:** M. B. Raft, T. S. H. Jørgensen, J. Renneberg, C. F. Lassen, R. K. Jacobsen, A. Jorgensen, M. B. Jørgensen, M. Osler

**Affiliations:** 1 Department for Social Medicine, Bispebjerg and Frederiksberg Hospital, Copenhagen, Denmark; 2 Center for Clinical Research and Prevention, Bispebjerg and Frederiksberg Hospital, Copenhagen, Denmark; 3 Institute of Public Health, University of Copenhagen, Copenhagen, Denmark; 4 Department of Clinical Medicine, University of Copenhagen, Copenhagen, Denmark; 5 Psychiatric Center Copenhagen, Mental Health Services, Copenhagen, Denmark

**Keywords:** Major depressive disorder, Geographical inequities, Neighborhood SEP, Healthcare utilization, Antidepressants

## Abstract

**Purpose:**

This study examines geographical distribution of depression recognized in primary versus secondary care across Danish municipalities and the association between municipality-level socioeconomic indicators and depression.

**Methods:**

The study was based on data from the Danish registers. We followed ~ 5,5 million individuals aged > 18 years during the period 2012–2023 for antidepressant prescriptions (reflecting primary care) or hospital-recorded depression diagnoses (reflecting secondary care). Hazard ratios (HR) of (1) antidepressants and hospital diagnoses across municipalities and (2) antidepressants and hospital diagnoses across four municipality-level socioeconomic indicators, were calculated. Lee’s L statistics were applied to assess spatial associations and median hazard ratios were estimated as a measure of between-municipality variation.

**Results:**

We found an overall positive spatial association between the two outcomes. Most municipality-level indicators were associated with higher antidepressant prescription rates, after adjustment for individual factors, strongest for high proportions of short education and transfer payments (HR 1.16 (95% CI 1.10-1.23 and 1.17 (95% CI 1.10-1.23), respectively). Associations with hospital-recorded depression were largely attenuated, after adjustment for individual factors. Between-municipality variation was modest (MHR 1.14 for antidepressant prescriptions and 1.19 for depressions diagnoses).

**Conclusion:**

We showed that municipalities with socioeconomic disadvantage had higher rates of antidepressant prescriptions. However, a large part of the between-municipality variation remains unexplained, highlighting the need to address contextual determinants beyond individual-level factors, even within a universal healthcare system.

**Supplementary Information:**

The online version contains supplementary material available at https://doi.org/10.1007/s00127-026-03055-x.

## Introduction

Depression is the most frequent mental disorder worldwide, with a lifetime risk of 12–16% [1]. Despite effective diagnostic tools and treatments, many individuals neither seek mental health care nor receive adequate treatment [2]. Delayed or inadequate treatment might worsen the prognosis with regards to disease severity, treatment response and risk of relapse [3–5]. Under-recognition and undertreatment have been reported in several studies [6–8], and might be a result of barriers such as stigma [9] and low awareness of mental health [10] or structural barriers like limited access, availability, or quality of local services [11]. At the individual level, having low socioeconomic position (SEP) is associated with both the risk of under-recognition [7] and under-treatment [12] of depression. Additionally, studies have shown that living in areas with socioeconomic disadvantage is associated with higher prevalence of depressive symptoms [13, 14], but lower usage of mental health services [15, 16], although the evidence on the latter is mixed. Nonetheless, these findings indicate that certain areas might be underserved due to geographic or social disadvantages. Previous literature has suggested several mechanisms explaining the association between social context and healthcare utilization. A widely studied mechanism is limited access to healthcare in deprived areas, as also described in the inverse care law, which states that the availability of medical care tends to vary inversely to the need of the population served [17]. However, other theories point to factors beyond healthcare supply. For example, Kirby and Kaneda propose that disadvantage becomes an ‘‘emergent characteristic’’ of these areas, reducing the resident’s ability to obtain adequate healthcare [18]. At the general practitioner level, a higher proportion of patients with low socioeconomic status may provide more complex health issues, which require more time and resources than what is typically feasible within a standard consultation [19]. Such conditions have also been found to increase the risk of GP burnout [20], potentially lowering the quality of primary care [21] in these areas.

The aim of this study was two-fold. First, we wanted to assess the geographical variance of modest to severe depression recognized within primary and secondary care between municipalities of Denmark. Second, we wanted to evaluate the association between local socioeconomic resources and depression diagnosis in these two settings.

## Method

### Study population

**Fig. 1:**
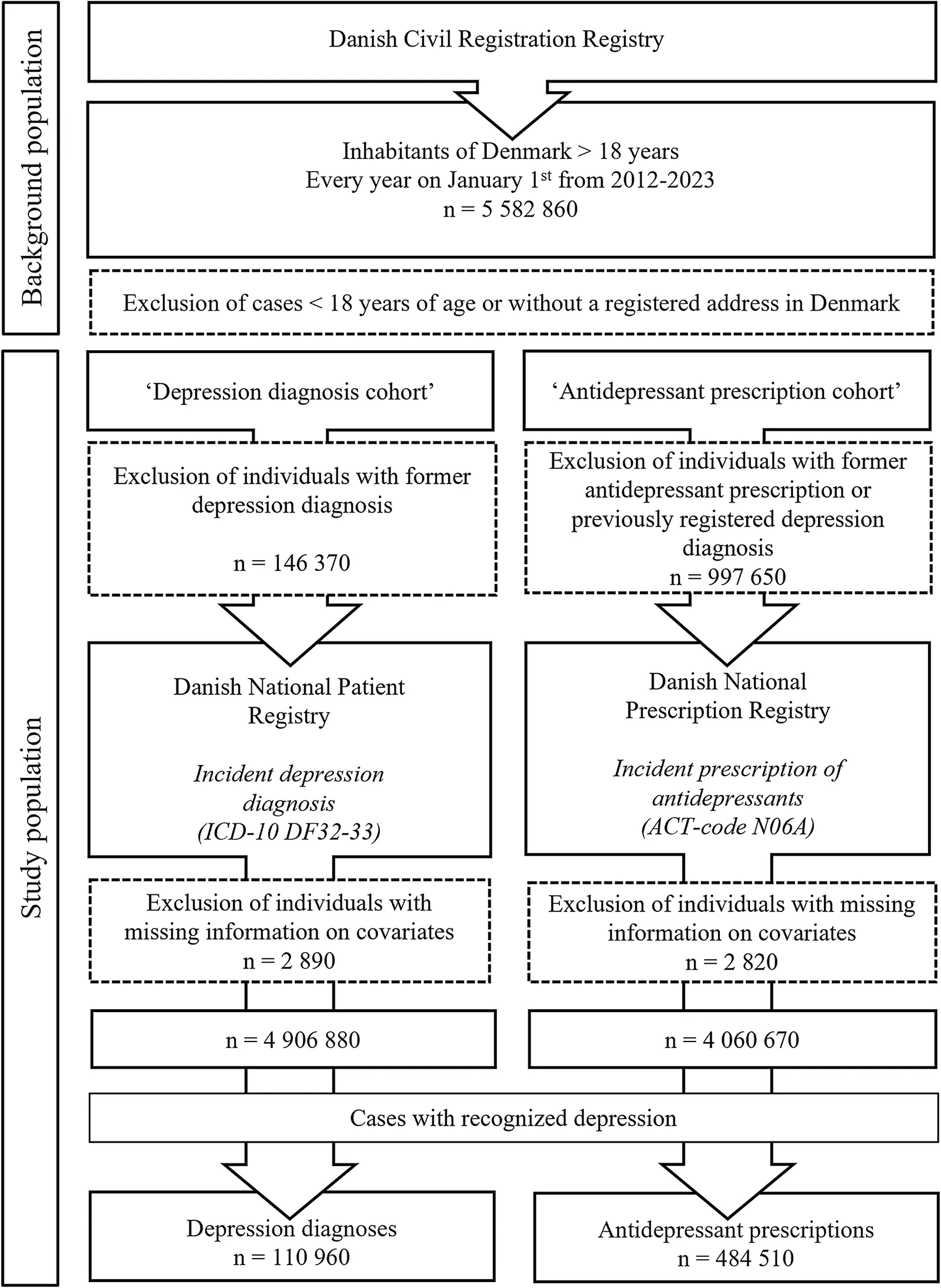
Flowchart illustrating the formation of the study population

The study population was identified in the Danish Civil Registration System using personal identification number (CPR), which enables linkage of recorded individual-level information in all registers [27]. The cohort consisted of persons who were > 18 years and living in Denmark at the start of the follow-up period (January 1 st, 2012) or at their 18 years’ birthday, whichever occurred last (*n* = 5,582,860). We constructed two cohorts: one to study the outcome ‘antidepressant prescriptions’ reflecting treatment in primary care and a second to study the outcome ‘hospital-recorded diagnosis of depression’ reflecting treatment in secondary care. For simplicity, throughout this article, we will refer to the first cohort as the ‘antidepressant prescription cohort’ and the latter as the ‘depression diagnosis cohort’. To detect cases with incident contact to primary care for depression treatment, we excluded individuals with a previously registered depression diagnosis (registered before 2012 or baseline, whichever occurred last) and/or a former prescription of antidepressants (prescribed before 2012 or baseline, whichever occurred last) from the ‘antidepressant prescription cohort’ (n = 997,650). To detect cases with incident contact to secondary care for depression treatment, we excluded individuals with a previously registered depression diagnosis (registered before 2012 or baseline, whichever occurred last) from the ‘depression diagnosis cohort’ (n = 146,370). The ‘depression diagnosis cohort’ included individuals who had a previous prescription of antidepressants, as the outcome was intended to detect cases who may not have responded to treatment in primary care setting. Individuals who had missing information on covariates or who had no registered address in Denmark were excluded. See Figure 1. All counts of individuals, events and PYR have been rounded to nearest 10.

### Exposure

A description of the study setting can be found in Online Resource (Supplementary Methods). Data on municipality of residence were obtained from the Danish Civil Registration System. Municipalities with < 1500 inhabitants (e.g. smaller islands) (*n* = 1) was merged with the neighboring municipality, resulting in a total of 97 municipalities. Information on municipality-level socioeconomic indicators was retrieved from Statistics Denmark’s online statistical database at baseline (year 2012). To assess the relative contribution of different dimensions of municipality-level socioeconomic resources and to enhance comparability with other studies, we used four different single variables of disadvantage, as described elsewhere in previous research on area-level SEP and health [30, 31]. We used the following measures at municipality-level, categorized into quartile groups (low, medium low, medium high, high): Average equivalized disposable family income, the proportion of the population (age > 25 years) with short education (defined as primary school as highest attainment or missing education data), and the proportion of the population on public welfare benefits (not including State Educational Grants). Additionally, we included the socioeconomic index that is defined by the Ministry of the Interior and Health of Denmark and used in the municipal equalization system to estimate the municipality expenditure needs and tax bases [32]. A lower value indicates a more affluent municipality. A list of all the variables in the index can be found in Online Resource 1 (Supplementary Table 1).

### Outcomes

The primary outcomes were first-time prescription of antidepressive medication (reflecting primary care treatment) or first-time hospital-recorded depression diagnosis (reflecting secondary care treatment). Individuals with a depression diagnosis were identified in the Danish National Patient Registry, that contains information on in- and out-patient hospital contacts [57] using the International Classification of Diseases and Related Health Problems (ICD-10) codes F32 (‘Depressive episode’) and F33 (‘Recurrent depressive disorder’). Only primary diagnoses were included. To identify individuals with depression who had been treated in primary care, we used data on first antidepressant prescription from the Danish National Prescription Registry [33] based on the Anatomic Therapeutical Chemical (ATC) classification system code N06A. This group comprises all antidepressants regardless of mode of action. Besides ATC codes, the register includes indication codes. To explore risk of misclassification from antidepressants prescribed for other indications (e.g. chronic pain or anxiety), we performed sensitivity analyses using the indication code “Against depression”.

### Covariates

All covariates were retrieved at baseline (2012) or at the year of study entry (i.e. whenever they turned 18). Sex was defined as male or female. For descriptive statistics, age was grouped into four categories: 17–44, 45–64, 65–79, and 80 + years. The highest achieved educational attainment was categorized according to the International Standard Classification of Education (ISCED 2011) into short (primary school), medium (high school, vocational education) and long (higher education). Equivalized disposable family income was categorized into low-, medium- or high-income groups based on tertile distributions. We used the Employment Categorization Module (AKM) to identify individuals outside of work force by grouping recipients of public welfare benefits together based on the classifications from SOCIO13: “Unemployed more than half of the year”, “Disability Pension”, “Sick Leave/Other Leave” and “Social Security”. Cohabitation status was defined as living alone or cohabiting. Immigrant status was defined as individuals originating from non-western countries. The comorbidities included four major somatic chronic illnesses (Cancer (ICD-10 codes C00-C97), Ischemic Heart Disease (ICD-10 codes I20-I25), Chronic Obstructive Pulmonary Disease (COPD) (ICD-10 code J44) and Diabetes Mellitus (ICD-10 codes E10-E14 and ACT-code A10)) and all previously recorded psychiatric conditions (ICD-10 codes F00-F99) except depression.

### Missing data

In the depression diagnosis cohort, 2,890 individuals had missing data (570 for income and cohabitation and 2,320 for welfare benefits and pensioner status). In the antidepressant prescription cohort, 2,820 individuals were missing (570 for income and cohabitation and 2,250 for welfare benefits and pensioner status). These individuals were excluded. Individuals missing on education (individuals born before 1920 and older immigrants [34]) were combined into the short education group based on the distribution of the remaining covariates. See Fig. 1.

### Data analyses

Two survival models were conducted. For all analyses, the cause specific hazard ratios were calculated separately using cox proportional hazards regression. Individuals were followed from age at study entry until the age of first registration of outcome, emigration, death, or end of follow-up (31st December 2022), whichever came first. Municipality and aggregated socioeconomic data were entered as time-dependent variables, meaning that individuals who moved to another municipality changed exposure status according to the new residence. The models were stratified by birth cohort (10-year intervals), thereby adjusting for calendar effects.

In the first model, we estimated HR’s of the two outcomes for each municipality. Using an effect parametrization method, HR’s were calculated relative to the country mean. To visualize the variation across municipalities, we constructed maps using the software QGIS illustrating the delineations of the municipalities and the corresponding HR’s of each municipality. To assess the bivariate spatial association between HR’s for antidepressants and HR’s for hospital diagnoses, we applied Lee’s L statistics [35]. First, we calculated the global Lee’s L to estimate the overall spatial patterning of the two measures. Positive values (0 to 1) indicate a positive spatial association (similar clustering of high or low values), while negative values (–1 to 0) indicate a negative spatial association (inverse clustering of high and low values). Second, to detect areas where the local association deviated from the global association, we computed local Lee’s L statistics, and constructed a map of concordance or discordance between the two variables (e.g., high-high, low-low, high-low, low-high patterns). Statistical significance was assessed usingpermutation tests with 4,999 permutations, which is recommended for tests at a 95% confidence level [36]. Spatial weights were based on Queen contiguity and isolated units (islands) were connected manually.

In the second model, we estimated HR’s for the outcomes using one of the four indicators as exposure. To account for the hierarchical structure of the data, with individuals (level 1) nested in municipalities (level 2), we addressed a multilevel design using a frailty model in which we incorporated a cluster-specific random effect of municipalities, allowing us to assess variation between municipalities. These models are also known as shared frailty models because all subjects that are nested within the same cluster share the same frailty [37]. To quantify the magnitude of this frailty, reflecting a general contextual effect (GCE), we estimated the median hazard ratios (MHR). This estimation can be used as a measure of the difference in hazards when comparing individuals with identical covariates from different clusters. An MHR of 1 indicates no contextual effect, whereas higher values reflect greater between-cluster variation in hazards. When the frailty is assumed to follow a gamma distribution, the MHR is given by the upper quantile of an F(2$\:\sigma\:$^−2^, 2$\:\sigma\:$^−2^), where $\:\sigma\:$ = the variance of the gamma distribution [38]. First, the MHR was estimated for the null model, to assess the GCE of the municipalities. Next, we included a municipality-level indicator of SEP, to estimate the specific contextual effect (SCE) of that variable. Finally, we fitted fully adjusted models with individual-level covariates, to account for compositional differences. This step-wise approach, allowed us to evaluate (1) whether there was variation at all between the municipalities, and, if so, the magnitude of it (i.e. the GCE) and (2) determine how much of the variation is explained by the context (i.e. the SCE) versus the composition (i.e. the individual-level covariates) [39].

To estimate the competing risk of death due to possible geographical variation in mortality rates, a hazard regression analysis for risk of death was conducted to explore if municipalities with lower depression rates had higher mortality rates. This approach was used as the Nelson-Aalen cumulative hazard regression is not appropriate for designs with time-varying exposures. Results are shown in Online Resource 1 (Supplementary Table 4).

In all models, a significance level of 5% was applied. All statistical analyses were conducted using SAS version 9.4. Spatial statistics were conducted in R Studio version 2025.05.0 + 496.

## Results

The study included 4,060,670 individuals (36,432,000 person-years at risk) in the antidepressant prescription cohort and 4,906,880 individuals (46,412,740 person-years at risk) in the depression diagnosis cohort. During follow-up, 484,510 individuals received antidepressants, and 110,960 had a hospital-recorded depression diagnosis. Distributions of individual and municipal characteristics by outcome are shown in Tables 1 and 2.

**Table 1:** Incidence rate by individual-level characteristics

Depression diagnosis cohort				Antidepressant prescription cohort				
**Individual-level variables**	**No. of individuals**	**No. of events**	**Total PYR**	**IR per 100 000 PYR (95% CI)**	**No. of individuals**	**No. of events**	**Total PYR**	**IR per 100 000 PYR (95% CI)**
**Total**	4 906 880	110 960	46 412 740	239 (238-241)	4 060 670	484 510	36 432 000	1330 (1326-1334)
**Sex**								
Female	2 460 580	68 990	23 316 010	296 (294-298)	1 947 281	266 580	17 337 30	1 540 (1 530-1 540)
Male	2 446 300	41 960	23 096 780	182 (180-183)	2 113 667	217 930	19 094 70	1 140 (1 140-1 150)
**Age group**								
17-44 years	2 529 900	69 820	23 106 570	302 (300-304)	2 250 760	241 900	19 313 70	1 253 (1 248-1 258)
45-64 years	1 446 080	23 630	15 294 490	155 (153-157)	1 111 130	118 640	11 283 800	1 051 (1 045-1 057)
65-79 years	712 040	13 550	6 752 709	201 (197-204)	547 760	91 760	4 990 120	1 839 (1 827-1 851)
+ 80 years	218 860	3 960	1 258 900	314 (304-324)	151 030	32 220	845 010	3 813 (3 771-3 854)
**Cohabitation status**								
Living alone	1 285 490	35 900	11 476 950	312 (310-316)	975 410	134 390	8 256 070	1 243 (1 239-1 247)
Cohabiting	3 621 390	75 060	34 935 790	215 (213-216)	3 085 260	350 130	28 175 30	1 628 (1 619-1 636)
**Disposable family income**								
Lowest third (T1)	1 552 360	48 110	13 945 250	345 (342-348)	1 284 070	199 270	10 804 00	1 844 (1 836-1 853)
Middle third (T2)	1 676 320	38 060	16 191 760	235 (233-237)	1 384 810	166 750	12 715 30	1 311 (1 305-1 318)
Highest third (T3)	1 678 190	24 790	16 275 740	152 (150-154)	1 391 790	118 490	12 911 670	918 (913-923)
**Receiving transfer payments**								
Yes	464 910	20 120	46 255 70	435 (429-441)	258 870	54 550	2 308 180	2 363 (2 343-2 383)
No	4 441 970	90 840	41 787 180	217 (216-219)	3 801 800	429 960	34 123 20	1 260 (1 256-1 264)
**Pensionist**								
Yes	1 004 970	18 310	8 602 660	213 (210-216)	750 990	128 130	6 233 720	2 056 (2 044-2 066)
No	3 901 920	92 650	37 810 080	245 (244-247)	3 309 680	356 380	30 198 80	1 180 (1 176-1 184)
**Education level**								
Short	2 016 700	56 720	16 512 440	344 (341-346)	1 684 960	223420	12 758 50	1 751 (1 744-1 758)
Medium	1 938 620	38 430	19 972 590	192 (191-194)	1 578 280	187860	15 609 80	1 204 (1 198-1 209)
Long	951 570	15 810	9 927 710	159 (157-161)	797 420	73230	8 063 770	908 (902 - 915)
**Immigrant status**								
Non-western origin	352 360	10 470	3 194 530	328 (322-334)	293 840	38 290	2 425 230	1 579 (1 563-1 595)
**Comorbidity**								
Number of chronic somatic diseases (HD, DM, cancer, COPD)								
1	436 340	9 200	3756070	245 (240 - 250)	304 820	52 270	2 506 800	2 090 (2 070 – 2 100)
2	56 980	1 300	396340	329 (311 - 346)	34 550	7 350	227 580	3 230 (3 160 – 3 300)
> 3	4 950	130	28390	465 (386 - 544)	2 720	630	14 010	4 480 (4 130 – 4 830)
Any previously registered psychiatric diagnosis								
Yes	312 690	20 110	2685490	748 (738 – 759)	162 050	35 650	1 180 560	3 019 (2 988 - 3 051)

Baseline sociodemographic characteristics for the study population according to hospital-recorded depression diagnosis and antidepressant prescriptions. Counts of individuals, events and PYR have been rounded to nearest 10. Abbreviations: IR: Incidence Rate, PYR: Person Years at Risk, HD: Heart Disease, DM: Diabetes Mellitus, COPD: Chronic Obstructive Pulmonary Disease

**Table 2:**
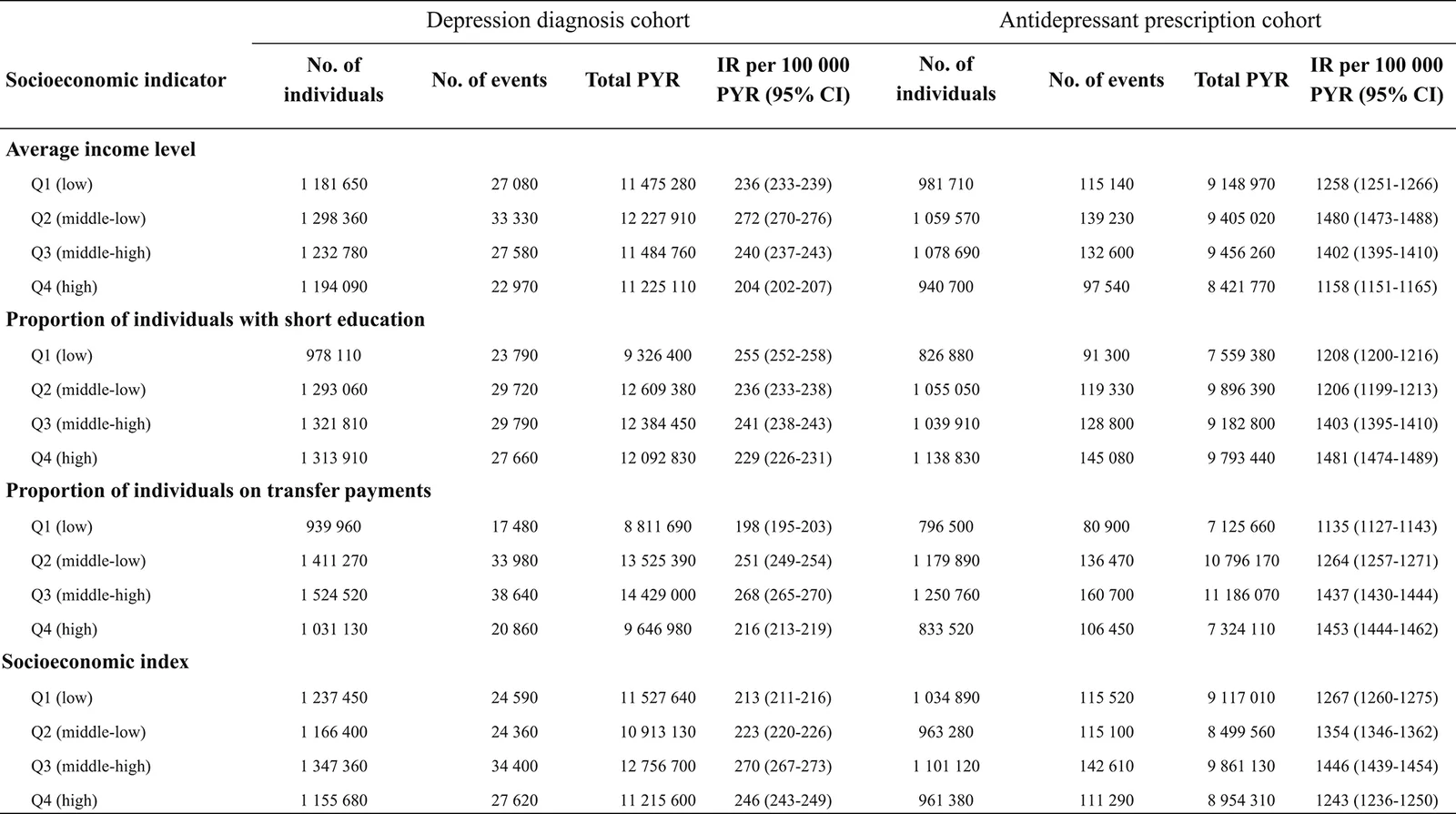
Incidence rate by municipality-level socioeconomic indicators

Baseline sociodemographic characteristics for the study population according to hospital-recorded depression diagnosis and antidepressant prescriptions. Counts of individuals, events and PYR have been rounded to nearest 10. Abbreviations: IR, Incidence Rate; PYR, Person Years at Risk; HD, Heart Disease; DM, Diabetes Mellitus; COPD, Chronic Obstructive Pulmonary Disease

### Geographical variation of depression

**Fig. 2:**
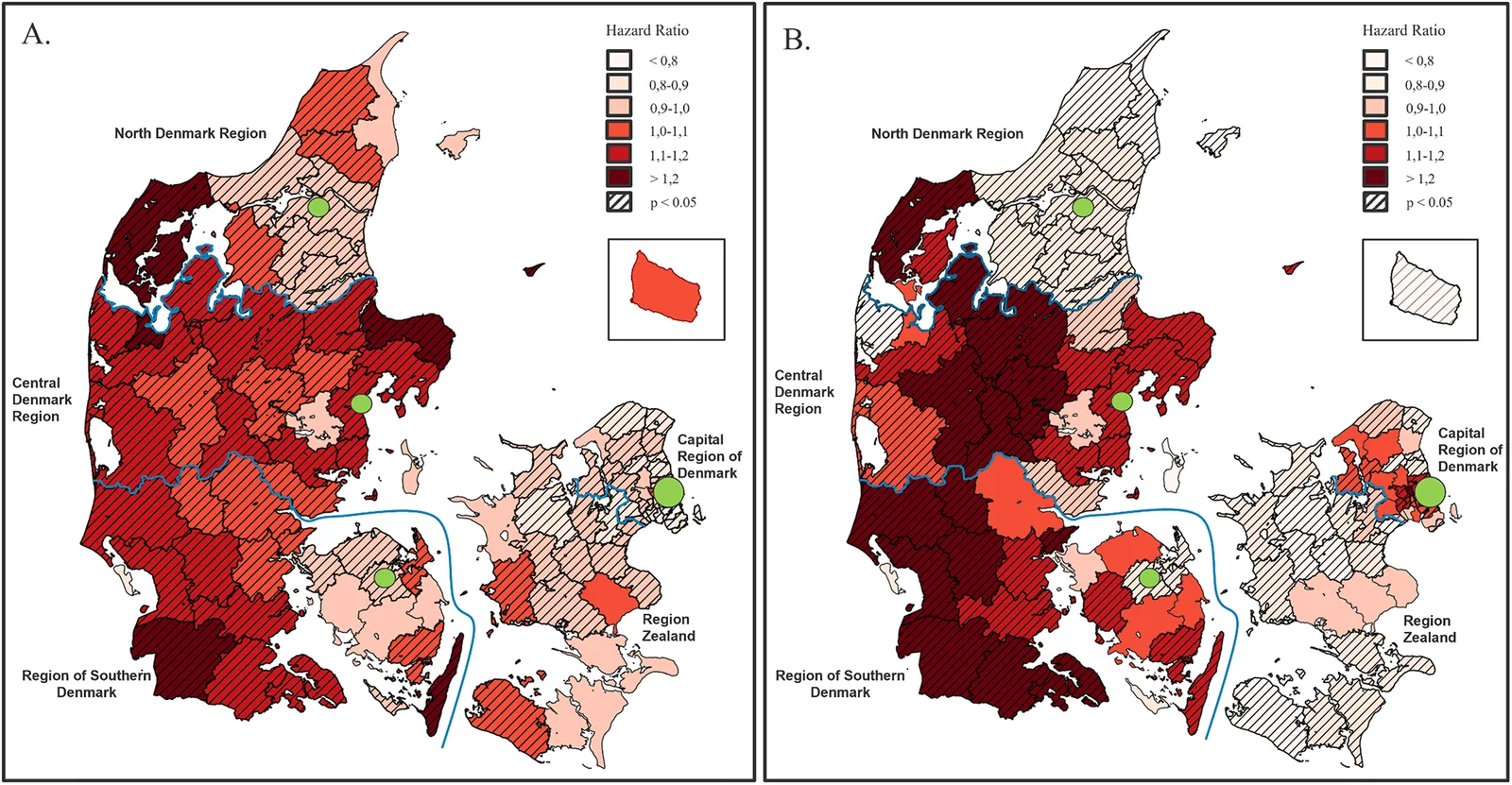
Hazard rations for each municipality adjusted for individual-level factors. Municipalities of Denmark with corresponding HR for incident antidepressant prescription (**A**) and hospital diagnosis of depression (**B**) compared to country average. Municipalities with a significantly lower or higher HR than country average are scratched. Largest cities are shown by green dots. Blue lines indicate the delineations of Denmark’s five administrative regions. Abbr.: HR: Hazard ratio

**Fig. 3:**
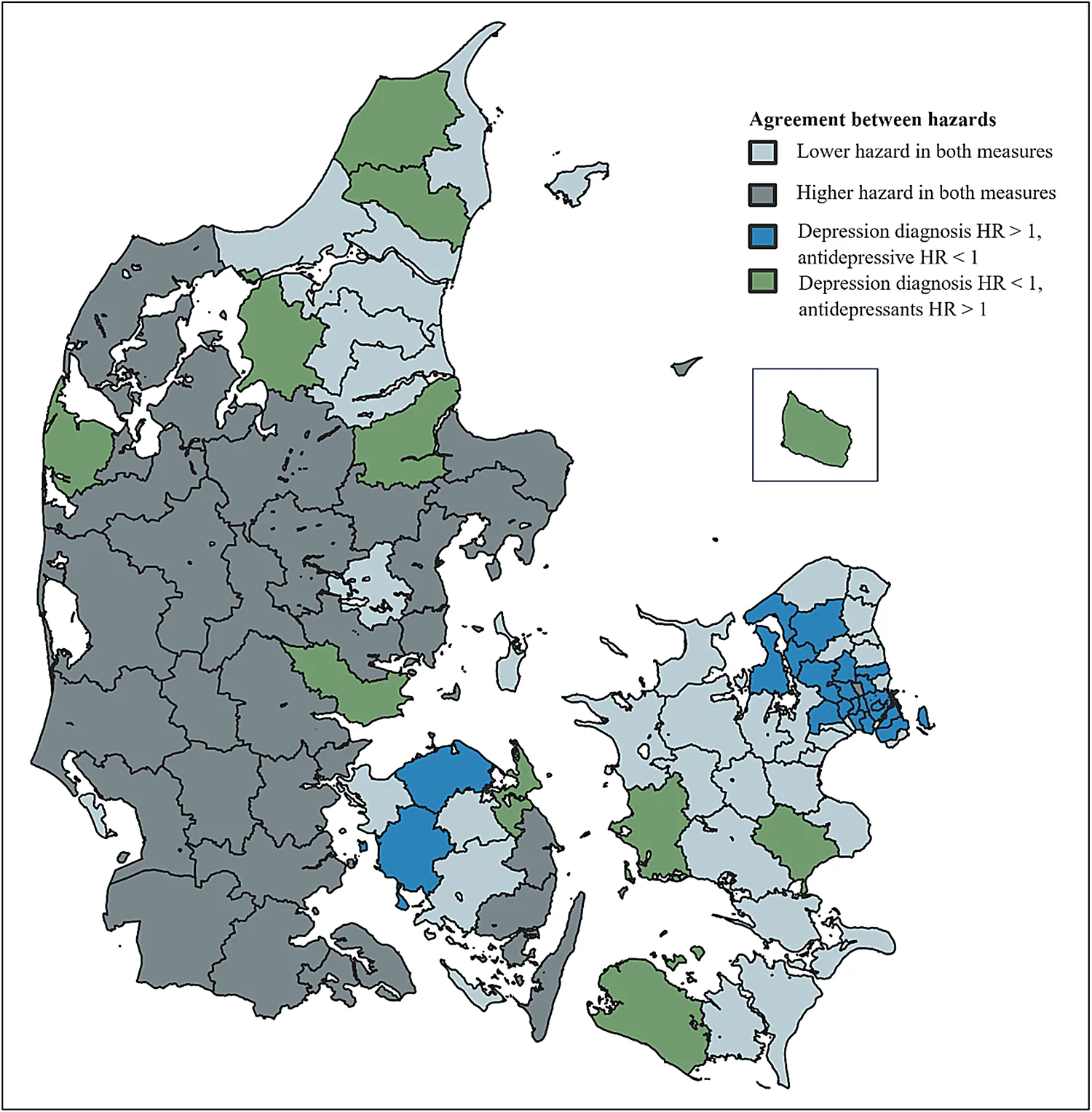
Agreement between HR’s of antidepressant prescriptions (primary sector contacts) and depression diagnosis (hospital contacts). Municipalities with elevated HR compared to country mean for antidepressant prescription, but not correspondingly elevated HR for hospital diagnosis are marked in green, whereas municipalities with elevated HR for hospital diagnosis compared to country average, but not correspondingly for antidepressant prescriptions are marked in blue. Abbreviations: HR: Hazard Ratio

In the adjusted models, the hazards ratios for both antidepressant prescriptions and depression diagnoses varied across municipalities. In adjusted models, 81 of 97 municipalities showed significantly different HR’s for antidepressant prescriptions (38 higher, 43 lower) compared to the country average. For depression diagnoses, 67 municipalities differed (35 higher, 32 lower). A full list of HR’s by municipality are provided in Online Resource 1 (Supplementary Tables 2 A and 2B). When mapping the adjusted HR’s for antidepressant prescriptions and hospital diagnoses across municipalities (Fig. 2), we observed that areas with higher HR’s for one outcome generally showed higher ratios for the other. In some municipalities, however, the patterns diverged, with HR’s being higher for prescriptions but lower for hospital diagnoses, or vice versa (Fig. 3). Applying Lee’s spatial statistic confirmed a statistically significant positive global spatial association between the two outcomes (Global Lee’s L = 0.41, p < 0.001), indicating that municipalities tended to exhibit similar patterns for both measures, with clusters of high HR’s for antidepressant use coinciding with high HR’s for hospital diagnoses, and likewise for low values. Mapping local Lee’s L indicated that these clusters of high-high or low-low were concentrated in certain regions of Denmark, as illustrated in Online Resource 1 (Supplementary Fig. 1).

### Municipality-level socioeconomic indicators

#### Antidepressant prescriptions

In multilevel models we found that all the municipality-level socioeconomic indicators, besides Socioeconomic Index, were associated with higher HRs of antidepressant prescriptions, after adjusting for individual risk factors. The strongest associations were observed for municipalities with high proportions of individuals with short education and medium high proportion on transfer payments, where individuals had higher HR of antidepressant prescriptions (respectively, 1.16 (95% CI 1.10-1.23) and 1.17 (95% CI 1.10-1.23)), compared to municipalities with low proportions (reference). HR’s for antidepressant prescriptions by municipality-level socioeconomic indicators are shown in Table 3. The MHR for the null model was 1.14, indicating moderate between-municipality variation. In the unadjusted models with municipality-level indicators as exposure, the MHR was reduced to 1.11 for average income level, 1.10 for proportion of individuals on transfer payments, 1.10 for proportion of individuals with short education and 1.13 for the socioeconomic index. In models adjusted for individual-level variables the MHR was 1.11 for the socioeconomic index and 1.10 for all other exposures.

**Table 3:**
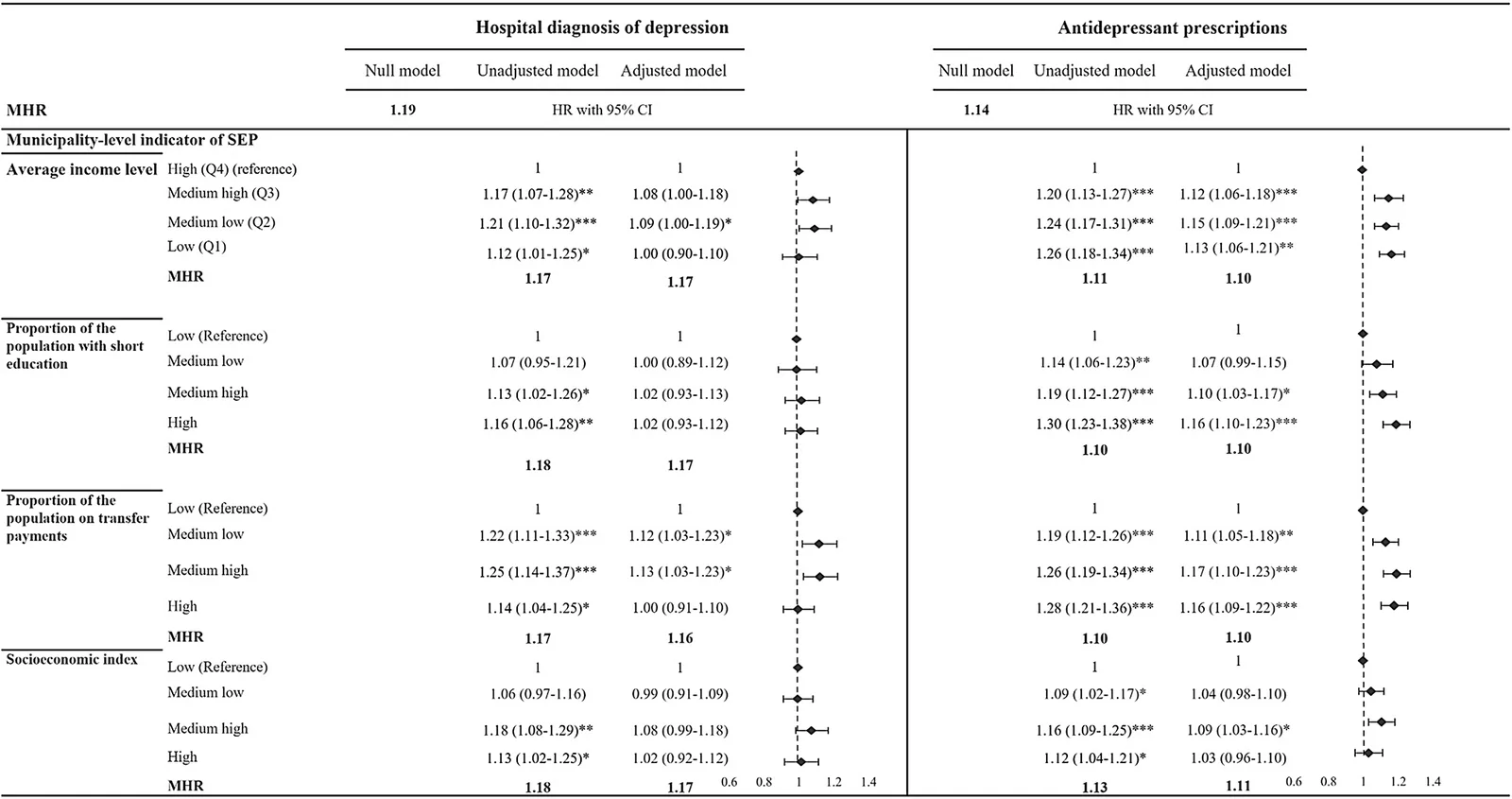
Multilevel survival analysis examining HR of hospital diagnosis of depression or antidepressive treatment by municipality-level socioeconomic indicators

Unadjusted and adjusted multilevel Cox regression with individual and municipality- level socioeconomic measures as fixed effects and municipality of residence as random effect, using age as time scale. Depression diagnosis at hospital or antidepressive treatment are outcomes of interest. Hazard ratios with 95% CI interval are listed and for the adjusted analyses illustrated with forest plots. Adjusted models includes following covariates: Gender, cohabitation status, education level, immigrant status, income level, labor market affiliation and comorbidities. Median hazard ratios for null model, models with specific contextual exposure (unadjusted model) and fully adjusted models (adjusted model). *P-value <0,05. **P-value <0,001. ***P-value <0,0001. Abbreviations: HR: Hazard Ratio; CI: Confidence Interval; MHR: Median Hazard Ratio.

### Hospital diagnosis of depression

In the unadjusted model, all the municipality-level indicators were significantly associated with higher HRs of hospital-recorded depression diagnosis. After adjusting for individual-level variables, the associations became insignificant, except for individuals living in municipalities with medium high (HR 1.13 (95% CI 1.03-1.23)) and medium low (HR 1.12 (95% CI 1.03-1.23)) proportions of the population on transfer payments and individuals living in municipalities with medium low income level (HR 1.09 (95% CI 1.00-1.19)). when compared to municipalities with low proportions. However, these associations were only borderline significant. HR’s for depression diagnosis by municipality-level socioeconomic indicators are shown in Table 3. The MHR for the null model was 1.19. In the unadjusted models with municipality-level indicators as exposure, the MHR was reduced to 1.17 for average income level, 1.17 for proportion of individuals on transfer payments, 1.18 for proportion of individuals with low education and 1.18 for the socioeconomic index. Including individual-level variables, reduced the MHR further to 1.17 for proportion of individuals with low education and 1.17 for the socioeconomic index, whereas the others were unchanged.

## Discussion

In this nationwide Danish cohort study, we found a heterogenous geographical distribution of first-time primary care treatment (measured by antidepressant prescriptions) and first-time secondary care treatment (measured by hospital diagnoses) for depression. We found a positive spatial association between antidepressant rates and hospital diagnosis rates, indicating an overall concordance between the two outcomes (areas with high rates of antidepressant prescription had corresponding high rates of hospital diagnoses and vice versa). We also found that municipalities with lower socioeconomic indicators had higher rates of antidepressant prescriptions. The strongest association was observed between municipalities with high proportions of individuals with short education or on transfer payments, where the antidepressant prescription rate was 16% and 17% higher than municipaltities with low proportions. The estimated MHR for the null model was 1.14 and 1.19 for antidepressants and hospital diagnoses, respectively. This can be interpreted as the general contextual effect of municipalities, before considering specific contextual exposures [39]. After including the specific contextual exposures at municipality-level, the MHR decreased slightly for both outcomes, but the reduction was overall small, suggesting that the specific contextual exposures explained only some of between-municipality variation.

Considering that depression is a major public health problem, it is important to examine individual differences in utilization of mental health services based on residential context. Thus, uncovering patterns of differential utilization is of importance when planning the allocation of municipality-based health resources. Throughout the past decades, the number of studies investigating area-level determinants of mental health has increased extensively, with several reviews concluding that there is evidence of an association between living in socioeconomically disadvantaged areas and depressive symptoms [40–42]. However, fewer studies have focused on differences in utilization of mental health services, based on residential location [43, 44]. In relation to our study, Crump et al. also found an increased use of antidepressants in lower SEP areas of Sweden [45], whereas in another study, Bocquier et al. found that living in deprived areas were associated with lower odds of receiving antidepressive treatment [46]. Also, in contrast to our study, Gannedahl et al. found higher use of specialized care in municipalities with low SEP [47]. However, a critical difference in this study compared to the current study is that it included a study population that had already been diagnosed with depression in general practice. Studies investigating what is often referred to as “neighborhood effects” on health outcomes are faced with a number of methodological challenges, especially with regards to the conceptualization of context [48–50]. As such, issues have been raised about the inconsistencies in the results that are depending on the areal unit of choice (a phenomenon known as the modifiable areal unit problem (MAUP) addressed by Openshaw in 1984 [51]). Another issue arises when arbitrary neighborhood boundaries fail to reflect individuals’ true geographic context (referred to as the uncertain geographic context problem (UGCoP) as devised by Kwan et al. [52]). Thus, a number of reviews conclude that clear methodologies are needed when delineating the areal context of interest [40, 49, 53, 54]. In this study we used a territorial, fixed geographical unit as spatial scale (e.g. as described by Chaix et al. [49]) to examine if the resources within this context influenced mental healthcare pathways. The rationale for using municipalities as context, is that they represent entities with a great part of autonomy in prioritizing, and planning welfare benefits, including local healthcare services that ought to prevent disease and promote health and rehabilitation. As financing these benefits are based on the income taxes of the residents, it is likely that these varies from one municipality to another, even though the equalization system ensures a minimum of services in all municipalities. Underpinning this, a recent report from The National Institute of Public Health of Denmark concluded that it varies which health promoting and preventative initiatives the municipalities are offering to its residents [55]. These variations might not be problematic, if they correspond to the needs of the residents in the municipality. By using individual SEP as proxies of healthcare need at the individual-level, the findings from our study, however, could indicate, that the variation in depression is not fully explained by differences in the composition of the residents in the municipality.

For hospital diagnoses, reflecting cases with a higher level of severity, or patients with insufficient response to treatment initiated in primary care settings, we found no association with municipality indicators of SEP. Correspondingly, the reduction in MHR was overall small or non-existing, indicating that neither municipality-level SEP nor individual-level factors, fully explained the between-municipality variation. Several possible mechanisms may explain the findings. First, the depression diagnosis cohort had fewer cases than the prescription cohort, which may reduce statistical power. However, given the large sample and adjusted HRs near 1, this likely does not explain the null findings.

Secondly, including individual factors enables an estimation of how much is explained by the composition of the municipality’s population. Thus, the attenuation of the initial estimates indicate that the association was mainly due to clustering of individuals with low SEP in low-SEP municipalities. The persistent MHR > 1, however, indicates unmeasured contextual factors, that were beyond the scope of this study. We speculate that e.g. accessibility or proximity of hospitals in the area, might play a role.

For antidepressants, reflecting contacts in primary care setting, we found an association with municipality indicators of SEP beyond the individual (compositional) factors. The largest reduction in MHR was seen for transfer payments and short education, both accounting for approximately 3.5% of the variation. In fully adjusted models, an additional small reduction was observed, indicating that the individual-level covariates accounted for some variation, however, substantial residual contextual heterogeneity remained. This means, that even though the socioeconomic indicators were statistically significant, they explained only a small proportion of the between-municipality variation. Thus, we speculate that the geographic variation we observe, could be explained by systematic differences in healthcare processes between areas, e.g. treatment preferences in the population, different thresholds for prescribing antidepressants or for referrals to non-pharmacological treatment options e.g. psychologists. These speculations, however, needs to be further examined in future research.

### Strengths and limitations

This study has several strengths that deserve to be emphasized. One major strength is the longitudinal study design based on comprehensive national registers with minimal data gaps in both the individual and the municipality variables. By using linkages through personal identification numbers, this study provides a national scope on geographic inequities. Furthermore, by using time-varying variables for municipality of residence, we were able to update exposure variables if the individuals moved from one municipality to another, thereby increasing the validity of the results.

However, the study also has important limitations that need to be addressed. First, even though we considered factors that both impact need for mental healthcare and area of residency, there is still a risk of residual confounding. Although potential confounding was handled to some extend by adjusting the models for individual-level factors that might drive self-selection into certain areas and impact the need for mental healthcare, administrative data may not capture all relevant factors e.g. unobserved familial risk factors and other lifestyle related factors. Considering e.g. genetic dispositions would be highly relevant, as families often tend to live in adjacency to each other. Furthermore, using individual risk factors based on administrative data as a measure of mental healthcare need, may not accurately reflect true need.

Second, the study relies on socioeconomic indicators for the municipalities measured at baseline, meaning that we did not account for changes in the municipal social resources over time. Including time-varying exposure variables would be computationally unmanageable as it would affect the data size enormously. To estimate the impact of this limitation, we considered how many municipalities changed quartile group for the socioeconomic indicators throughout the study period. The majority of the municipalities stayed within the same quartile throughout the study period (*n* = 86 for proportion on transfer payments, *n* = 76 for proportion with short education and *n* = 54 for average income level), however the remaining municipalities fluctuated between the adjacent quartile group.

Third, as mentioned previously, using antidepressant prescriptions as proxy for treatment of depression in the primary sector has some limitations. One limitation is potential misclassification from using antidepressants without indication codes, as this may include treatment for other conditions (e.g., anxiety or chronic pain). Indication codes are applied by the physician who prescribes the medicine, and overall, the prescription register accurately captures indications. However, some issues regarding a large degree of missing and non-specific recorded indication codes exists [56]. Thus, restricting cases to those with the indication code “Against depression” could, on the other hand, potentially lead to some cases being false negatives. Additionally, this restriction would reduce the sample size and thus the statistical power. These considerations combined with the consistent results from the sensitivity analyses supported inclusion of all antidepressants in this study (See Online resource 1 (Supplementary Table 3)). Another limitation of using antidepressants with relevance to this study, is that treatment in primary care setting includes a wide range of treatment options besides antidepressive medication, e.g. psychotherapy/psychologist consultations, and furthermore, we were not able to distinguish between antidepressants prescribed at the GP and antidepressants prescribed at a practicing psychiatrist upon referral from the GP. Thus, it is important to acknowledge that the dimensions and the different levels of primary care treatment are not captured in this study. 

And finally, when dealing with multiple municipalities, incorporating non-proportional effects would make the models overly comprehensive. Even though accounting for non-proportionality could have provided nuanced insights, it was not the focus of this study. Therefore, we adopted a simpler analytical approach, thereby restricting HR’s between municipalities to be constant at all ages.

## Conclusion

We found a positive spatial association between primary and secondary care contacts for depression. Furthermore, we found an association between municipality-level socioeconomic indicators and antidepressant prescriptions, independent of individual factors. However, only a small part of the variation between municipalities was explained, suggesting that unmeasured contextual factors related to residential location influence treatment pathways. Attention should continuously be directed at contextual factors operating beyond individual-level factors, even in a high-income country with a universal healthcare system.

## Supplementary Information

Below is the link to the electronic supplementary material.


Supplementary Material 1


## Data Availability

The individual-level data used for this study cannot be shared due to restrictions on access to data stored at Statistics Denmark. Researchers outside Denmark may obtain data access by collaborating with Danish research institutes that are authorized by Statistics Denmark. The aggregated data on municipalities are publicly available and can be generated from the StatBank at Statistics Denmark (https://www.statbank.dk/statbank5a/default.asp?w=1920) and from The Ministry of the Interior and Health of Denmark (https://www.noegletal.dk/noegletal/ntStart.html).
