# Supplementary material for: Geographical variation and area-level socioeconomic disadvantage in recognized depression: A nationwide register-based Danish cohort study
Source: Soc Psychiatry Psychiatr Epidemiol. 2026 Apr 29;61(8):1425–37. doi: 10.1007/s00127-026-03055-x (PMC13522088; doi:10.1007/s00127-026-03055-x)
Supplement: Supplementary file 1 [file 127_2026_3055_MOESM1_ESM.docx]

### Online Resource 1 (supplementary material) for the article

### Geographical variation and area-level socioeconomic disadvantage in recognized incident depression: A nationwide register-based Danish cohort study

MB Raft (1,2), TSH Jørgensen (2,3), J Renneberg (1), CF Lassen (1), RK Jacobsen (2), (4, 5), MB Jørgensen (4,5), M Osler (2).

(1) *Department for Social Medicine, Bispebjerg and Frederiksberg Hospital, Copenhagen, Denmark*
(2) *Center for Clinical Research and Prevention, Bispebjerg and Frederiksberg Hospital, Copenhagen, Denmark*
(3) *Institute of Public Health, University of Copenhagen, Denmark*
(4) *Department of Clinical Medicine, University of Copenhagen, Denmark*
(5) *Psychiatric Center Copenhagen, Mental Health Services, Copenhagen, Denmark*

**List of content**

**Supplementary Method** Description of study setting

**Supplementary Table 1** Variables in the Socioeconomic Index

**Supplementary Table 2A+2B** Adjusted Hazard Ratios for antidepressants and depression diagnosis for each municipality compared to country mean

**Supplementary Table 3** Sensitivity analysis using indication codes for antidepressants

**Supplementary Table 4** Competing risk of death

**Supplementary Figure 1** Map of local bivariate spatial association

**Corresponding author:** Marie Birkvig Raft

**E-mail:** marie.birkvig.raft@regionh.dk

## Setting

Denmark is a small country located in northern Europe with 5.9 million inhabitants in 2024 [1]. The Danish welfare model includes a universal and tax-funded health care system, operating through three administrative and political levels; the national level (state), the regional level (5 regions) and the municipal level (98 municipalities). While the state is responsible for the national legislations and health care planning, the regions are responsible for managing and prioritizing health care services in the primary sector, including general practitioners and private practicing specialists, and the secondary sector including public hospitals. The municipalities are responsible for the planning and organization of local health care services to warrant disease prevention, health promotion and rehabilitation [2]. The prevention programs are not mandatory by law [3] and should be based on the needs of the populations in the municipality [4].

| **Supplementary Table 1** Variables in the Socioeconomic Index used in the equalization act to calculate socioeconomic expenditure need per inhabitant | |
| --- | --- |
| **Variables** | **Weight** |
| Number of 20–59-year-olds outside work force | 0,19 |
| Number of 25–49-year-olds without vocational education | 0,13 |
| Number of affordable housing units | 0,06 |
| Number of psychiatric patients diagnosed within a 10-year period at psychiatric hospitals | 0,02 |
| Number of public family housing units | 0,12 |
| Number of children in families where guardians have low education | 0,09 |
| Number of individuals over 65 years living alone | 0,03 |
| Number of persons with low income in three out of four years | 0,05 |
| Number of disabled persons | 0,04 |
| Number of immigrants and descendants | 0,06 |
| Number of 20–59-year-olds wage earners with basic level skills | 0,04 |
| Number of children of single providers | 0,02 |
| Number of children who have moved three times | 0,03 |
| Distance to workplaces | 0,02 |
| Regional unemployment rate | 0,02 |
| Number of 45-64-year-olds with limited work experience | 0,05 |
| Population density | 0,01 |
| Low average life expectancy relative to the municipality with the highest life expectancy | 0,02 |
| Sum of weighted criteria’s | 1,00 |

| **Supplementary Table 2A** Adjusted Hazard Ratios for antidepressants for each municipality compared to country mean | | | | |
| --- | --- | --- | --- | --- |
| **Municipality** | **P-value** | **Hazard Ratio** | **95% CI Lower** | **95% CI Upper** |
| Aabenraa | <.0001 | 1.1835 | 1.1535 | 1.2143 |
| Aalborg | <.0001 | 0.9180 | 0.9042 | 0.9321 |
| Aarhus | <.0001 | 1.0345 | 1.0220 | 1.0471 |
| Albertslund | 0.0080 | 0.9472 | 0.9101 | 0.9859 |
| Allerød | <.0001 | 0.8442 | 0.8042 | 0.8862 |
| Assens | 0.9423 | 1.0012 | 0.9692 | 1.0342 |
| Ballerup | 0.2297 | 0.9818 | 0.9527 | 1.0117 |
| Billund | <.0001 | 1.0894 | 1.0478 | 1.1325 |
| Bornholm | 0.1088 | 1.0260 | 0.9943 | 1.0586 |
| Brøndby | <.0001 | 0.8807 | 0.8495 | 0.9130 |
| Brønderslev | 0.2515 | 1.0202 | 0.9859 | 1.0556 |
| Dragør | <.0001 | 0.8220 | 0.7715 | 0.8758 |
| Egedal | 0.0011 | 0.9438 | 0.9116 | 0.9771 |
| Esbjerg | <.0001 | 1.1260 | 1.1049 | 1.1475 |
| Faaborg-Midtfyn | 0.7418 | 1.0048 | 0.9764 | 1.0342 |
| Fanø | 0.6238 | 0.9709 | 0.8630 | 1.0924 |
| Favrskov | <.0001 | 1.1117 | 1.0784 | 1.1460 |
| Faxe | 0.7929 | 1.0047 | 0.9704 | 1.0401 |
| Fredensborg | 0.0028 | 0.9479 | 0.9151 | 0.9818 |
| Fredericia | <.0001 | 1.0707 | 1.0407 | 1.1016 |
| Frederiksberg | <.0001 | 0.7267 | 0.7094 | 0.7444 |
| Frederikshavn | 0.7050 | 1.0050 | 0.9793 | 1.0314 |
| Frederikssund | <.0001 | 0.9344 | 0.9046 | 0.9652 |
| Furesø | 0.5267 | 1.0115 | 0.9764 | 1.0479 |
| Gentofte | <.0001 | 0.9009 | 0.8762 | 0.9263 |
| Gladsaxe | <.0001 | 0.9482 | 0.9232 | 0.9739 |
| Glostrup | <.0001 | 0.9043 | 0.8644 | 0.9461 |
| Greve | <.0001 | 0.9363 | 0.9070 | 0.9665 |
| Gribskov | <.0001 | 0.8652 | 0.8354 | 0.8960 |
| Guldborgsund | 0.0016 | 0.9586 | 0.9338 | 0.9841 |
| Haderslev | <.0001 | 1.1599 | 1.1298 | 1.1908 |
| Halsnæs | 0.5671 | 0.9893 | 0.9534 | 1.0265 |
| Hedensted | <.0001 | 1.0669 | 1.0350 | 1.0998 |
| Helsingør | <.0001 | 0.9278 | 0.9023 | 0.9541 |
| Herlev | 0.7220 | 0.9929 | 0.9546 | 1.0327 |
| Herning | <.0001 | 1.0567 | 1.0336 | 1.0803 |
| Hillerød | <.0001 | 0.9326 | 0.9038 | 0.9624 |
| Hjørring | <.0001 | 1.0618 | 1.0359 | 1.0884 |
| Holbæk | <.0001 | 0.9156 | 0.8920 | 0.9398 |
| Holstebro | <.0001 | 1.0838 | 1.0551 | 1.1133 |
| Horsens | <.0001 | 1.0968 | 1.0726 | 1.1215 |
| Hvidovre | <.0001 | 0.8973 | 0.8707 | 0.9248 |
| Høje-Taastrup | <.0001 | 0.8663 | 0.8392 | 0.8942 |
| Hørsholm | <.0001 | 0.8564 | 0.8169 | 0.8978 |
| Ikast-Brande | <.0001 | 1.1489 | 1.1143 | 1.1846 |
| Ishøj | <.0001 | 0.8924 | 0.8527 | 0.9340 |
| Jammerbugt | 0.0207 | 0.9611 | 0.9293 | 0.9940 |
| Kalundborg | 0.0188 | 0.9645 | 0.9359 | 0.9940 |
| Kerteminde | 0.0235 | 1.0489 | 1.0065 | 1.0931 |
| Kolding | <.0001 | 1.0999 | 1.0764 | 1.1240 |
| København | <.0001 | 0.7598 | 0.7517 | 0.7681 |
| Køge | 0.0228 | 0.9680 | 0.9412 | 0.9955 |
| Langeland | <.0001 | 1.2867 | 1.2242 | 1.3524 |
| Lejre | 0.0003 | 0.9250 | 0.8865 | 0.9653 |
| Lemvig | <.0001 | 1.2008 | 1.1510 | 1.2527 |
| Lolland | <.0001 | 1.0911 | 1.0600 | 1.1232 |
| Lyngby-Taarbæk | <.0001 | 0.9179 | 0.8900 | 0.9467 |
| Mariagerfjord | 0.2451 | 0.9812 | 0.9504 | 1.0131 |
| Middelfart | 0.0183 | 0.9590 | 0.9262 | 0.9929 |
| Morsø | <.0001 | 1.2659 | 1.2156 | 1.3182 |
| Norddjurs | <.0001 | 1.2392 | 1.2020 | 1.2776 |
| Nordfyns | 0.0217 | 0.9558 | 0.9196 | 0.9934 |
| Nyborg | 0.8626 | 1.0032 | 0.9676 | 1.0401 |
| Næstved | <.0001 | 0.9295 | 0.9076 | 0.9520 |
| Odder | 0.0001 | 1.0897 | 1.0431 | 1.1384 |
| Odense | <.0001 | 0.9039 | 0.8898 | 0.9183 |
| Odsherred | 0.0004 | 0.9366 | 0.9032 | 0.9712 |
| Randers | <.0001 | 1.1609 | 1.1378 | 1.1845 |
| Rebild | <.0001 | 0.9188 | 0.8820 | 0.9572 |
| Ringkøbing-Skjern | <.0001 | 1.1375 | 1.1081 | 1.1677 |
| Ringsted | <.0001 | 0.9140 | 0.8802 | 0.9490 |
| Roskilde | <.0001 | 0.8549 | 0.8341 | 0.8763 |
| Rudersdal | <.0001 | 0.9385 | 0.9099 | 0.9680 |
| Rødovre | <.0001 | 0.8641 | 0.8343 | 0.8951 |
| Samsø | 0.4447 | 1.0402 | 0.9401 | 1.1510 |
| Silkeborg | <.0001 | 1.1018 | 1.0781 | 1.1261 |
| Skanderborg | 0.0016 | 1.0470 | 1.0176 | 1.0772 |
| Skive | <.0001 | 1.1143 | 1.0828 | 1.1467 |
| Slagelse | 0.5681 | 1.0068 | 0.9837 | 1.0304 |
| Solrød | 0.0052 | 0.9339 | 0.8902 | 0.9797 |
| Sorø | 0.0003 | 0.9296 | 0.8933 | 0.9675 |
| Stevns | <.0001 | 0.8891 | 0.8487 | 0.9314 |
| Struer | <.0001 | 1.2511 | 1.2013 | 1.3029 |
| Svendborg | <.0001 | 1.0630 | 1.0351 | 1.0917 |
| Syddjurs | <.0001 | 1.1785 | 1.1433 | 1.2149 |
| Sønderborg | <.0001 | 1.1605 | 1.1341 | 1.1874 |
| Thisted | <.0001 | 1.2505 | 1.2155 | 1.2866 |
| Tårnby | <.0001 | 0.8794 | 0.8499 | 0.9100 |
| Tønder | <.0001 | 1.2818 | 1.2438 | 1.3209 |
| Vallensbæk | <.0001 | 0.7994 | 0.7527 | 0.8490 |
| Varde | <.0001 | 1.1354 | 1.1042 | 1.1675 |
| Vejen | <.0001 | 1.1678 | 1.1334 | 1.2033 |
| Vejle | 0.0944 | 1.0173 | 0.9971 | 1.0379 |
| Vesthimmerlands | <.0001 | 1.0785 | 1.0443 | 1.1138 |
| Viborg | <.0001 | 1.0830 | 1.0602 | 1.1063 |
| Vordingborg | 0.0032 | 0.9545 | 0.9254 | 0.9846 |
| Ærø | 0.2603 | 0.9552 | 0.8819 | 1.0346 |

| **Supplementary Table 2B** Adjusted Hazard Ratios for depression diagnoses for each municipality compared to country mean | | | | | |
| --- | --- | --- | --- | --- | --- |
| **Municipality** | **P-value** | **Hazard Ratio** | **95%CI Lower** | | **95%CI Upper** |
| Aabenraa | <.0001 | 1.3469 | 1.2804 | 1.4168 | |
| Aalborg | <.0001 | 0.7931 | 0.7666 | 0.8206 | |
| Aarhus | <.0001 | 1.2880 | 1.2591 | 1.3176 | |
| Albertslund | 0.0001 | 1.1606 | 1.0764 | 1.2514 | |
| Allerød | 0.0010 | 0.8285 | 0.7410 | 0.9264 | |
| Assens | 0.0031 | 1.1059 | 1.0346 | 1.1820 | |
| Ballerup | 0.0947 | 1.0541 | 0.9909 | 1.1213 | |
| Billund | <.0001 | 1.2023 | 1.1091 | 1.3034 | |
| Bornholm | <.0001 | 0.7908 | 0.7317 | 0.8547 | |
| Brøndby | 0.0397 | 0.9245 | 0.8578 | 0.9963 | |
| Brønderslev | 0.0018 | 0.8809 | 0.8134 | 0.9541 | |
| Dragør | 0.9249 | 0.9935 | 0.8679 | 1.1373 | |
| Egedal | 0.2301 | 1.0463 | 0.9718 | 1.1266 | |
| Esbjerg | <.0001 | 1.2085 | 1.1636 | 1.2552 | |
| Faaborg-Midtfyn | 0.9138 | 1.0035 | 0.9427 | 1.0682 | |
| Fanø | 0.8710 | 1.0221 | 0.7849 | 1.3311 | |
| Favrskov | <.0001 | 1.2141 | 1.1398 | 1.2932 | |
| Faxe | 0.2811 | 0.9585 | 0.8874 | 1.0353 | |
| Fredensborg | 0.6635 | 0.9833 | 0.9116 | 1.0607 | |
| Fredericia | 0.0001 | 1.1176 | 1.0557 | 1.1830 | |
| Frederiksberg | <.0001 | 0.8910 | 0.8505 | 0.9334 | |
| Frederikshavn | <.0001 | 0.8315 | 0.7814 | 0.8848 | |
| Frederikssund | 0.5243 | 0.9776 | 0.9116 | 1.0483 | |
| Furesø | 0.2607 | 1.0451 | 0.9678 | 1.1286 | |
| Gentofte | 0.0058 | 0.9175 | 0.8630 | 0.9754 | |
| Gladsaxe | 0.2418 | 1.0331 | 0.9783 | 1.0910 | |
| Glostrup | 0.2655 | 1.0516 | 0.9625 | 1.1491 | |
| Greve | <.0001 | 0.7659 | 0.7090 | 0.8272 | |
| Gribskov | 0.4550 | 1.0280 | 0.9561 | 1.1053 | |
| Guldborgsund | <.0001 | 0.8240 | 0.7751 | 0.8760 | |
| Haderslev | <.0001 | 1.1872 | 1.1245 | 1.2533 | |
| Halsnæs | 0.3104 | 1.0417 | 0.9626 | 1.1273 | |
| Hedensted | 0.5844 | 1.0189 | 0.9528 | 1.0896 | |
| Helsingør | <.0001 | 0.8645 | 0.8125 | 0.9198 | |
| Herlev | 0.0584 | 1.0802 | 0.9973 | 1.1700 | |
| Herning | <.0001 | 1.1849 | 1.1333 | 1.2388 | |
| Hillerød | 0.3004 | 0.9654 | 0.9031 | 1.0319 | |
| Hjørring | <.0001 | 0.8181 | 0.7698 | 0.8695 | |
| Holbæk | <.0001 | 0.8168 | 0.7698 | 0.8667 | |
| Holstebro | 0.0116 | 1.0757 | 1.0164 | 1.1385 | |
| Horsens | 0.0045 | 1.0696 | 1.0211 | 1.1205 | |
| Hvidovre | 0.7001 | 0.9880 | 0.9290 | 1.0507 | |
| Høje-Taastrup | 0.3112 | 0.9676 | 0.9077 | 1.0313 | |
| Hørsholm | 0.1474 | 0.9258 | 0.8341 | 1.0276 | |
| Ikast-Brande | <.0001 | 1.2295 | 1.1532 | 1.3108 | |
| Ishøj | 0.0546 | 0.9110 | 0.8284 | 1.0018 | |
| Jammerbugt | 0.0007 | 0.8739 | 0.8087 | 0.9445 | |
| Kalundborg | <.0001 | 0.8686 | 0.8119 | 0.9292 | |
| Kerteminde | 0.0057 | 0.8719 | 0.7911 | 0.9609 | |
| Kolding | 0.0008 | 1.0802 | 1.0324 | 1.1302 | |
| København | <.0001 | 0.9176 | 0.8988 | 0.9368 | |
| Køge | <.0001 | 0.8811 | 0.8275 | 0.9382 | |
| Langeland | 0.1256 | 1.0948 | 0.9750 | 1.2293 | |
| Lejre | 0.0279 | 0.8972 | 0.8146 | 0.9883 | |
| Lemvig | 0.0296 | 0.8890 | 0.7996 | 0.9884 | |
| Lolland | <.0001 | 0.7912 | 0.7353 | 0.8512 | |
| Lyngby-Taarbæk | 0.6830 | 1.0135 | 0.9503 | 1.0809 | |
| Mariagerfjord | <.0001 | 0.8631 | 0.8019 | 0.9289 | |
| Middelfart | 0.2262 | 0.9547 | 0.8857 | 1.0291 | |
| Morsø | 0.0001 | 1.1914 | 1.0905 | 1.3017 | |
| Norddjurs | <.0001 | 1.1690 | 1.0946 | 1.2484 | |
| Nordfyns | 0.5568 | 1.0248 | 0.9444 | 1.1121 | |
| Nyborg | 0.4595 | 1.0291 | 0.9538 | 1.1104 | |
| Næstved | 0.0653 | 0.9540 | 0.9074 | 1.0030 | |
| Odder | <.0001 | 1.2297 | 1.1240 | 1.3453 | |
| Odense | <.0001 | 0.8785 | 0.8502 | 0.9077 | |
| Odsherred | 0.0004 | 0.8616 | 0.7938 | 0.9353 | |
| Randers | 0.0036 | 0.9336 | 0.8915 | 0.9778 | |
| Rebild | <.0001 | 0.8188 | 0.7439 | 0.9013 | |
| Ringkøbing-Skjern | 0.0005 | 1.1094 | 1.0468 | 1.1756 | |
| Ringsted | <.0001 | 0.8031 | 0.7377 | 0.8743 | |
| Roskilde | 0.0001 | 0.9030 | 0.8572 | 0.9511 | |
| Rudersdal | 0.5750 | 0.9807 | 0.9160 | 1.0499 | |
| Rødovre | 0.3397 | 0.9659 | 0.8995 | 1.0372 | |
| Samsø | 0.8494 | 0.9768 | 0.7663 | 1.2450 | |
| Silkeborg | <.0001 | 1.3197 | 1.2646 | 1.3771 | |
| Skanderborg | 0.0001 | 1.1251 | 1.0600 | 1.1941 | |
| Skive | <.0001 | 1.1737 | 1.1050 | 1.2467 | |
| Slagelse | <.0001 | 0.8874 | 0.8428 | 0.9343 | |
| Solrød | 0.0236 | 0.8807 | 0.7890 | 0.9831 | |
| Sorø | <.0001 | 0.7992 | 0.7289 | 0.8764 | |
| Stevns | 0.2086 | 0.9379 | 0.8486 | 1.0365 | |
| Struer | 0.0049 | 1.1380 | 1.0401 | 1.2451 | |
| Svendborg | 0.0002 | 1.1084 | 1.0491 | 1.1711 | |
| Syddjurs | <.0001 | 1.1641 | 1.0889 | 1.2444 | |
| Sønderborg | <.0001 | 1.2433 | 1.1863 | 1.3030 | |
| Thisted | <.0001 | 1.2049 | 1.1324 | 1.2820 | |
| Tårnby | 0.4584 | 0.9733 | 0.9062 | 1.0454 | |
| Tønder | <.0001 | 1.3214 | 1.2420 | 1.4059 | |
| Vallensbæk | 0.2011 | 0.9220 | 0.8140 | 1.0442 | |
| Varde | <.0001 | 1.2168 | 1.1477 | 1.2900 | |
| Vejen | <.0001 | 1.2980 | 1.2212 | 1.3796 | |
| Vejle | 0.8992 | 1.0028 | 0.9607 | 1.0467 | |
| Vesthimmerlands | 0.0132 | 0.9077 | 0.8408 | 0.9800 | |
| Viborg | <.0001 | 1.2706 | 1.2189 | 1.3245 | |
| Vordingborg | <.0001 | 0.8327 | 0.7754 | 0.8943 | |
| Ærø | 0.9145 | 1.0098 | 0.8451 | 1.2066 | |

## Antidepressants with indication codes for depression

When including only antidepressant prescriptions with an indication code ‘Against depression’, the number of cases was reduced to 107,337 individuals (≈22%) in the antidepressant prescription cohort. However, even though the associations diminished, they remained significant for most of the indicators except for ‘low average income level’ and ‘medium low proportion of population on transfer payments’, in which case the associations became insignificant (See Online Resource 1 (Supplementary Table 3)). In the spatial analyses, the direction and significance of the HR remained unchanged in 66 municipalities. In 25 municipalities the association became insignificant and in 6 municipalities the association became significant when only including cases of antidepressant prescriptions with indication codes for depression.

Considering that a great part of the reduction of cases with antidepressant prescriptions were due to indication codes being missing, we concluded that overall, for the purpose of this study, the sensitivity analyses demonstrated acceptable robustness of the results using antidepressants without indication codes, which was preferred to avoid unwarranted exclusion of cases from the study resulting in a reduction of statistical power.

| **Supplementary Table 3** Sensitivity analysis using indication codes for antidepressants | | | |
| --- | --- | --- | --- |
| **Municipality-level socioeconomic**  **indicator** | | **Antidepressive treatment** | |
|  |  | Without indication codes | With indication codes |
|  |  | HR with 95% CI | |
| **Average income level** | | | |
|  | High (Q4) (reference) | 1 | 1 |
|  | Medium high (Q3) | 1.10 (1.03-1.17)** | 1.05 (1.00-1.09)* |
|  | Medium low (Q2) | 1.13 (1.07-1.19)*** | 1.08 (1.03-1.13)* |
|  | Low (Q1) | 1.1 (1.03-1.17)* | 1.04 (0.98-1.09) |
| **Proportion of the population with short education** | | |  |
|  | Low (Reference) | 1 | 1 |
|  | Medium low | 1.05 (0.98-1.13) | 1.02 (0.97-1.08) |
|  | Medium high | 1.08 (1.02-1.15)* | 1.06 (1.01-1.14)* |
|  | High | 1.15 (1.09-1.21)*** | 1.09 (1.04- 1.14)** |
| **Proportion of the population on transfer payments** | | |  |
|  | Low (Reference) | 1 | 1 |
|  | Medium low | 1.08 (1.02-1.15)** | 1.03 (0.98-1.08) |
|  | Medium high | 1.14 (1.07-1.20)*** | 1.09 (1.04-1.14)** |
|  | High | 1.13 (1.06-1.19)*** | 1.05 (1.00-1.10)* |
| **Socioeconomic index** | |  |  |
|  | Low (Reference) | 1 | 1 |
|  | Medium low | 1.03 (0.97-1.09) | 1.01 (0.97-1.06) |
|  | Medium high | 1.08 (1.01-1.14)* | 1.05 (1.00-1.10)* |
|  | High | 1.01 (0.94-1.07) | 0.99 (0.94-1.04) |
| Sensitivity analyses using antidepressant prescriptions restricted to cases with a registered indication code “against depression” compared to using all cases. Adjusted multilevel Cox regression with individual and municipality level socioeconomic measures as fixed effects and municipality of residence as random effect. Hazard ratios with 95% CI interval are listed Adjusted for: Age, gender, cohabitation status, education level, income level, labor market affiliation and comorbidities. *P-value <0,05. **P-value <0,001. ***P-value <0,0001. Abbreviations: HR: Hazard Ratio; CI: Confidence Interval. | | | |

## Competing risk of death

In 14 out of 97 municipalities in the depression diagnosis cohort and 25 out of 97 municipalities in the antidepressant prescription cohort, we found that the HR for death without depression was significantly higher compared to country average and the HR for depression diagnoses or antidepressant prescriptions was significantly lower than country average, after adjustment for individual factors. Thus, the lower HR in these municipalities might reflect competing risk of death rather than lower occurrence or under-recognition of depression. The affected municipalities and corresponding HRs are listed in Online Resource 1 (Supplementary Table 4).

| **Supplementary Table 4** Competing risk of death | | | | | |  |  |  |
| --- | --- | --- | --- | --- | --- | --- | --- | --- |
|  | HR with 95% CI | | | | |  |  |  |
|  | Event of interest | | | Competing event  (death) | |  |  |  |
| **Antidepressive prescription** | | | | | |  |  |  |
| Municipality of Brøndby | 0.88 (0.85-0.91) | | | 1.12 (1.07-1.18) | |  |  |  |
| Municipality of Dragør | 0.82 (0.77-0.88) | | | 1.10 (1.02-1.18) | |  |  |  |
| Municipality of Egedal | 0.94 (0.91-0.98) | | | 1.11 (1.06-1.17) | |  |  |  |
| Municipality of Frederiksberg | 0.73 (0.71-0.74) | | | 1.11 (1.08-1.15) | |  |  |  |
| Municipality of Frederikssund | 0.93 (0.90-0.97) | | | 1.11 (1.07-1.16) | |  |  |  |
| Municipality of Gladsaxe | 0.95 (0.92-0.97) | | | 1.11 (1.07-1.15) | |  |  |  |
| Municipality of Greve | 0.94 (0.91-0.97) | | | 1.13 (1.08-1.19) | |  |  |  |
| Municipality of Gribskov | 0.87 (0.84-0.90) | | | 1.10 (1.06-1.15) | |  |  |  |
| Municipality of Guldborgsund | 0.96 (0.93-0.98) | | | 1.13 (1.10-1.17) | |  |  |  |
| Municipality of Helsingør | 0.93 (0.90-0.95) | | | 1.09 (1.05-1.13) | |  |  |  |
| Municipality of Hillerød | 0.93 (0.90-0.96) | | | 1.08 (1.04-1.13) | |  |  |  |
| Municipality of Holbæk | 0.92 (0.89-0.94) | | | 1.10 (1.06-1.14) | |  |  |  |
| Municipality of Hvidovre | 0.90 (0.87-0.92) | | | 1.11 (1.07-1.16) | |  |  |  |
| Municipality of Høje-Taastrup | 0.87 (0.84-0.89) | | | 1.13 (1.08-1.19) | |  |  |  |
| Municipality of Ishøj | 0.89 (0.85-0.93) | | | 1.12 (1.04-1.20) | |  |  |  |
| Municipality of Copenhagen | 0.76 (0.75-0.77) | | | 1.23 (1.21-1.25) | |  |  |  |
| Municipality of Lyngby-Taarbæk | 0.92 (0.89-0.95) | | | 1.14 (1.10-1.19) | |  |  |  |
| Municipality of Næstved | 0.93 (0.91-0.95) | | | 1.09 (1.05-1.22) | |  |  |  |
| Municipality of Odsherred | 0.94 (0.90-0.97) | | | 1.06 (1.02-1.11) | |  |  |  |
| Municipality of Roskilde | 0.86 (0.83-0.88) | | | 1.06 (1.03-1.10) | |  |  |  |
| Municipality of Rudersdal | 0.94 (0.91-0.97) | | | 1.07 (1.03-1.11) | |  |  |  |
| Municipality of Rødovre | 0.86 (0.83-0.90) | | | 1.16 (1.11-1.21) | |  |  |  |
| Municipality of Sorø | 0.93 (0.89-0.97) | | | 1.10 (1.04-1.16) | |  |  |  |
| Municipality of Tårnby | 0.88 (0.85-0.91) | | | 1.17 (1.12-1.22) | |  |  |  |
| Municipality of Vordingborg | 0.96 (0.93-0.98) | | | 1.11 (1.07-1.54) | |  |  |  |
| **Supplementary Table 4** (Continued) | | | | | |  |  |  |
|  | | HR with 95% CI | | | |  | |  |
|  | | | Event of interest | | Competing event  (death) |  |  | |
| **Depression diagnosis** | | | | | |  |  | |
| Municipality of Aalborg | | | 0.79 (0.77-0.82) | 1.02 (1.01-1.04) | |  |  |  |
| Municipality of Brøndby | | | 0.93 (0.86-1.00) | 1.10 (1.06-1.14) | |  |  |  |
| Municipality of Frederiksberg | | | 0.89 (0.85-0.93) | 1.06 (1.03-1.08) | |  |  |  |
| Municipality of Gentofte | | | 0.92 (0.86-0.98) | 1.03 (1.00-1.06) | |  |  |  |
| Municipality of Greve | | | 0.77 (0.71-0.83) | 1.10 (1.07-1.14) | |  |  |  |
| Municipality of Guldborgsund | | | 0.82 (0.78-0.88) | 1.07 (1.04-1.10) | |  |  |  |
| Municipality of Helsingør | | | 0.87 (0.81-0.92) | 1.04 (1.01-1.07) | |  |  |  |
| Municipality of Holbæk | | | 0.82 (0.78-0.87) | 1.04 (1.01-1.07) | |  |  |  |
| Municipality of Copenhagen | | | 0.92 (0.90-0.94) | 1.16 (1.15-1.18) | |  |  |  |
| Municipality of Lolland | | | 0.79 (0.74-0.85) | 1.18 (1.15-1.18) | |  |  |  |
| Municipality of Roskilde | | | 0.90 (0.86-0.95) | 1.04 (1.01-1.06) | |  |  |  |
| Municipality of Slagelse | | | 0.89 (0.84-0.93) | 1.08 (1.06-1.11) | |  |  |  |
| Municipality of Sorø | | | 0.80 (0.73-0.88) | 1.07 (1.03-1.11) | |  |  |  |
| Municipality of Vordingborg | | | 0.83 (0.78-0.90) | 1.08 (1.05-1.11) | |  |  |  |
| The listed municipalities have a higher hazard ratio of the competing event (death) compared to country average and a lover hazard ratio of the event of interest (antidepressive treatment or depression diagnosis) compared to country average. Abbreviations: HR, Hazard Ratio; CI, Confidence Interval. | | | | | |  |  |  |

**Supplementary Figure 1**: Bivariate Spatial Association Map

Bivariate spatial association map showing the local association between HR of antidepressants and HR of hospital diagnoses of depression across municipalities. Colors indicate the type of spatial association: dark red = high–high clusters (areas where HR’s for both measures are high in both the municipality and the neighboring municipalities), green = low–low clusters (areas where HR’s for both measures are high in both the municipality and the neighboring municipalities), lightblue = high–low (HR for antidepressants high, HR for hospital diagnoses low) and brown = (HR for antidepressants low, HR for hospital diagnoses high). Local Lee’s L statistics that were not at least 90% significant, were classified as not significant [5] (Grey areas). Abbreviations: HR: Hazard Ratio.

[1]	Statistics Denmark. Population Figures. Available from: https://www.dst.dk/en/Statistik/emner/borgere/befolkning/befolkningstal. 2024.

[2]	Ministry of Health, Healthcare Danmark. Healthcare in Denmark. 2017.

[3]	Schmidt M, Schmidt SAJ, Adelborg K, Sundbøll J, Laugesen K, Ehrenstein V, et al. The Danish health care system and epidemiological research: From health care contacts to database records. Clin Epidemiol 2019;11:563–91. https://doi.org/10.2147/CLEP.S179083.

[4]	Ministry of the Interior and Health. Municipal Equalization and General Grants 2008. 2007.

[5]	Esri. [ArcGIS Pro tool reference Documentation]. How bivariate spatial association works n.d. https://pro.arcgis.com/en/pro-app/3.4/tool-reference/spatial-statistics/how-bivariate-spatial-association-works.htm (accessed December 17, 2025).
